# Hybrid chitosan-lipid nanoparticles of green tea extract as natural anti-cellulite agent with superior *in vivo* potency: full synthesis and analysis

**DOI:** 10.1080/10717544.2021.1989088

**Published:** 2021-10-08

**Authors:** Sara A. Abosabaa, Mona G. Arafa, Aliaa Nabil ElMeshad

**Affiliations:** a Faculty of Pharmacy, Department of Pharmaceutics and Pharmaceutical Technology, The British University in Egypt (BUE), El Sherouk City, Egypt; b Chemotherapeutic Unit, Mansoura University Hospitals, Mansoura, Egypt; c Faculty of Pharmacy, Department of Pharmaceutics and Industrial Pharmacy, Cairo University, Cairo, Egypt; d Faculty of Pharmacy and Drug Technology, Department of Pharmaceutics, The Egyptian Chinese University, Cairo, Egypt

**Keywords:** Chitosan, green tea extract, lecithin, nanocarrier, ionic gelation, cellulite

## Abstract

The aim of this work is to exploit the advantages of chitosan (CS) as a nanocarrier for delivery of anti-cellulite drug, green tea extract (GTE), into subcutaneous adipose tissue. Primarily, analysis of herbal extract was conducted via newly developed and validated UPLC method. Ionic gelation method was adopted in the preparation of nanoparticles where the effect lecithin was investigated resulting in the formation of hybrid lipid-chitosan nanoparticles. Optimal formula showed a particle size of 292.6 ± 8.98 nm, polydispersity index of 0.253 ± 0.02, zeta potential of 41.03 ± 0.503 mV and an entrapment efficiency percent of 68.4 ± 1.88%. Successful interaction between CS, sodium tripolyphosphate (TPP) and lecithin was confirmed by Fourier-transform infrared spectroscopy, differential scanning calorimetry and X-ray diffraction. Morphological examination was done using transmission electron microscope and scanning electron microscope confirmed spherical uniform nature of GTE load CS-TPP nanoparticles. *Ex vivo* permeation study revealed permeability enhancing activity of the selected optimal formula due to higher GTE deposition in skin in comparison to GTE solution. Moreover *in vivo* study done on female albino Wistar rats carried out for 21 days proved successful potential anti-cellulite activity upon its application on rats’ skin. Histological examination showed significant reduction of adipocyte perimeter and area and fat layer thickness. Results of the current study demonstrated that the developed GTE-loaded CS-TPP nanoparticle comprised of chitosan and lecithin showed permeability enhancing activity along with the proven lipolytic effect of green tea represent a promising delivery system for anti-cellulite activity.

## 1. Introduction

Cellulite, referred as gynoid lipodystrophy, is a complex and multifactorial metabolic disorder of the subcutaneous fat layer characterized with skin dimpling and nodularity, designated as ‘orange-peel’ appearance. Nearly 85% of postpubertal females suffer from such condition especially in the abdominal, pelvic and lower limbs region (Rawlings, 2006). Its high percentage in female is governed by hormonal influence and the micro-architectural differences in the subcutaneous connective tissue in comparison to male (Rosenbaum et al., 1998). Changes in blood microcirculation and lymphatic disorders also contribute in the manifestation of cellulite (Kristiyani et al., 2018). Thus, it should be noted that cellulite is different from obesity, at which, the latter involves only adipocyte hypertrophy and hyperplasia and is unlimited to specific body parts.

Herbal extracts such as green tea (*Camellia sinensis*) have been extensively used in cosmeceutical preparations due to their high polyphenolic content exhibiting potent anti-oxidant activity. Application of herbal remedies worldwide have expanded owing to their therapeutic effect with relatively fewer side effects in comparison to other currently used treatments (Amer et al., 2020). Green tea extract (GTE) has an abundant number of benefits, including its anticarcinogenic and chemoprotective, and antibacterial activity (Balsaraf & Chole, 2015). Among these benefits, GTE can be employed as an anti-cellulite agent through multiple mechanisms. Primarily, the caffeine component acts through inhibition of the phosphodiesterase enzyme which results in subsequent elevation in cyclic adenosine monophosphate (cAMP) and increase activation of hormone sensitive lipase (HSL), thereby induce hydrolysis of triglyceride leading to cellulite reduction (Herman & Herman, 2013). Its effect can be synergized by the presence of other polyphenolic compounds including catechin (C), epicatechin (EC), and epigallocatechingallate (EGCG) through regulation of lipid anabolism and catabolism pathways (Moon et al., 2007). Despite the aforementioned described mechanisms of GTE as a potential agent in reducing cellulite, the use of whole GTE solely has not been examined as an anti-cellulite agent (Abosabaa et al., 2020). It is worth-mentioning that the utilization of GTE as a whole extract is more efficacious in comparison to using individual components based on the previous literature (Raederstorff et al., 2003; Chacko et al., 2010; Rasoanaivo et al., 2011). This is related to the fact that presence of additional constituents result in greater stability of EGCG in comparison to its isolated form, moreover, positive synergistic activity against cellulite is achieved (Chacko et al., 2010).

Stratum corneum (SC) is the main challenge in treating cellulite as it reduces the bioavailability of active pharmaceutical ingredients (API) in subcutaneous adipose tissue. On that account, incorporation of nanoparticles in topical formulations has been developed due to their ability in overcoming such barrier (Wu et al., 2010). The particle size (PS) highly influences permeation-ability of a formulation (Arafa & Ayoub, 2018), a PS of 600 nm will remain on the surface of SC, while a smaller size of 300 nm can penetrate into deeper skin layers (Danaei et al., 2018). Among the materials used in fabricating nanoparticles for topical delivery of drugs, chitosan (CS), a naturally occurring polysaccharide offers attractive characters that renders it an ideal topical delivery system. The hydrophilic and cationic (presence of positively charged amino group upon protonation) properties of this polymer imparts its permeability enhancing and bioadhesive characteristics (Fan et al., 2012). Most importantly, it can be easily cross-linked by polyanionic agents such as tripolyphosphate (TPP) forming chitosan nanoparticles via a technique referred as ionic gelation (Fan et al., 2012).

Despite the multiple advantages offered by such carriers as topical delivery system, yet, researches done in the field of cellulite are minimal (Abosabaa et al., 2020). This study reports the development of a novel bioactive loading drug delivery system in which GTE as a whole extract was loaded in polymeric nanocarrier, namely CS. Different parameters were investigated including entrapment efficiency percent (EE%), particle size (PS), polydispersity index (PDI) and zeta potential (ZP) to conclude the optimum formula. Moreover, microscopical analysis, differential scanning calorimetry (DSC), Fourier transform infrared (FTIR), x-ray diffraction (XRD), *in vitro* release and *ex vivo* permeation studies were performed for the selected formula. Thereafter, *in vivo* study was conducted to assess the potential anti-cellulite activity of the selected formula.

## 2. Materials and methods

### 2.1. Materials

Green tea leaves was purchased from Egyptian market. Chitosan (LMW 100 000–300 000 kDa), sodium tripolyphosphate, lecithin (soybean lecithin − 94% phosphatidylcholine), caffeine, catechin. epicatechin, and epigallocatechin gallate were purchased from Sigma-Aldrich Chemical Co. (St. Louis, USA). Acetic acid and Ethanol were supplied by Pio Chem, Cairo, Egypt. Sodium dihydrogen phosphate, sodium monohydrogen phosphate, acetonitirile, phosphoric acid and chloroform were purchased from Fisher Scientific (Loughborough, Leicestershire, UK). Cellophane membrane 12–14 kDa supplied by Spectrum Medical Inc, (Raleigh, North Carolina).

### 2.2. Methods

#### 2.2.1. Extraction procedure of green tea extract (GTE)

Fresh tea leaves were cleaned from impurities, sorted, washed, and dried at 100 °C. Rate of drying was observed by weighing the sample using analytical balance at specific time interval until a constant weight is attained. Extraction was performed using 20% ethanolic solution after crushing and sieving the dried leaves. Temperature of the extraction was maintained at 60 °C using temperature controlled water bath (Setyopratomo, 2014). Organic solvent was evaporated and dried residue of GTE was obtained.

#### 2.2.2. Phytochemical analysis

Ultra-performance liquid Chromatography (UPLC) (Thermo Fisher UPLC Model Ultimate 3000 (USA)), accompanied by photodiode array detector (PDA-3000RS, USA), an auto sampler (WPS3000TRS, USA) and gold hypersil C18 column was utilized for the separation and precise assay of four main components present in GTE namely caffeine, catechin (C), epicatechin (EC) and epigallocatechingallate (EGCG). Prior conducting UPLC analysis, preliminary UV-spectrophotometry study of total GTE was carried out and showed strongest signal around 270–278 nm, that was chiefly due to π-π* transition in caffeine and catechins (C, EC and EGCG) (Souto et al., 2010; Atomssa & Gholap, 2015). Direct determination, quantification and standardization of GTE was achieved through a newly developed chromatographic method. A mixture of orthophosphoric acid (0.1%) (solvent A) and acetonitrile (solvent B), were employed as the gradient mobile phase, which was degassed using water bath sonicator (Elmasonic S60H, Germany) prior conducting the analysis and delivered at a flow rate of 0.7 mL/min. A volume on 20 μL sample were injected using an autosampler each time with total run time of 60 min. Detector was set at 270 nm based on the preliminary UV analysis and three dimensions field detection was used. The gradient conditions were in the following manner: 0 min 5% solvent (B), 0 min to 14 min 15% solvent (B), 25 min to 53 min 35% solvent (B) and 53 min to 60 min 5% solvent (B). Validation of the developed analytical techniques was done on the basis of GTE fingerprint analysis, calibration curve linearity, accuracy, and precision, limit of detection (LoD) and quantification (LoQ) and bioanalytical validation.

#### 2.2.3. Preparation of GTE loaded CS-TPP nanoparticles:

GTE loaded CS-TPP nanoparticles were prepared by ionic gelation method reported by Calvo et al. (1997) with slight modifications. Addition of lipidic moiety, lecithin, was also carried out to produce hybrid lipid-chitosan nanoparticles. Several parameters were examined including EE%, PS, PDI and ZP.

Briefly, CS was added to 8 mL of 1% (v/v) acetic acid solution of pH 3 (pH meter-Jenway Model 3505- UK) to achieve a final formula composition of 0.25% in each sample. On the other hand, the cross-linker phase was composed of 2 mL distilled water containing different amounts of TPP to attain different CS: TPP mass ratios as demonstrated in Table 1. To the cross-linker phase, 10 mg of GTE was dissolved using water bath sonicator (Elmasonic S60 H, Elma Hans Schmidbauer GmbH, Singen, Germany). The effect of addition of lecithin was tested on different CS: TPP mass ratios (N4-N12) as shown in Table 1, the phospholipid was added in the cross-linker phase along with the GTE dissolved in 10% ethanolic solution. This mixture was subsequently added drop wisely into the CS solution under magnetic stirrer (Accuplate Hot plate stirrer, Lab Net International, Mexico) until a translucent nanoparticle suspension was formed and left to stir for one hour at 1000 rpm at ambient temperature. The resulted nanosuspension was sonicated using probe sonicator (Sonics Vibra Cell power 130 Watt, frequency 20 kHz, made by Sonics & Materials Inc, Newtown, CT) at 50 amplitude for 30 minutes. The formed nanoparticles were centrifuged by cooling centrifuge (Centurion Ltd. PRO-Research K241R- United Kingdom) at 15000 rpm at 4 °C for 45 minutes and supernatant was collected and used to assess drug entrapment efficiency.

**Table 1.** Composition of GTE-loaded CS-TPP nanoparticles with and without different additives.

Formulae	TPP concentration (%)	CS:TPP mass ratio	Amount of lecithin (mg)
N1	0.125	2: 1	–
N2	0.0625	4: 1	–
N3	0.042	6: 1	–
N4	0.125	2: 1	10
N5	0.125	2: 1	25
N6	0.125	2: 1	50
N7	0.0625	4: 1	10
N8	0.0625	4: 1	25
N6	0.0625	4: 1	50
N10	0.042	6: 1	10
N11	0.042	6: 1	25
N12	0.042	6: 1	50

#### 2.2.4. Evaluation of GTE-loaded CS-TPP nanoparticles

##### 2.2.4.1. Determination of entrapment efficiency percent (EE %)

The EE% was determined indirectly by detecting the amount of unentrapped GTE present in the collected supernatant using UV/Visible Spectrophotometer (Jasco – V-630- Japan) (Arafa et al., 2020). This is to test the total entrapment of only the desired investigated active constituents (caffeine, C, EGCG and EC) present in the GTE, as only these components give the same π → π* excitation emission at 270 nm as mentioned earlier. Blank nanoparticles were formulated under same conditions and their supernatant were used as control.

The EE% of GTE was calculated using equation:

(1) $$\text{EE\% }=\;\frac{\text{Total amount of GTE added}-\text{GTE unbound in supernatant}}{\text{Total amount of GTE added}}\times \;100$$

##### 2.2.4.2. Determination of particle size (PS), polydispersity index (PDI) and zeta potential (ZP)

All formulae freshly prepared and diluted were measured for average particle size, particle size distribution, and surface charge using Malvern Zetasizer (Malvern Instrument Ltd., UK) at 25 °C. Measurements were done in triplicates and the results were presented as mean ± standard deviation.

#### 2.2.5. Selection and characterization of the optimal formula

The selection of the optimized formulation was based on the optimum EE%, PS PDI and ZP. The entrapment of each the component, caffeine, C, EC and EGCG present in the selected formula was quantified by the ‘direct method’. This was done by collecting the residue remained after centrifugation, then dissolved in ethanol and vortex for 3 minutes to break down the nanoparticles (Tan et al., 2011). Ethanol was evaporated using Rotatory vacuum concentrator (CHRiST® Centrifuge: RVC 2–18 CD plus, Solvent trap: CT 02-50 SR, Vacuum Pump: DVP 2 C – TYRO 12- Germany) and residue was reconstituted using distilled water, filtered and EE% of each component was determined using UPLC developed method.

##### 2.2.5.1. Microscopic examination

###### 2.2.5.1.1. Transmission electron microscope (TEM)

The shape and morphology of the prepared nanoparticles were elucidated using TEM (H-600, Hitachi –Japan). Formula was diluted and one or two drops were placed on electron microscope (EM) grids (400- mesh carbon coated grids), then samples were stained using 1% phosphotungstic acid.

###### 2.2.5.1.2. Scanning electron microscope (SEM)

Illustration of the surface topography of the prepared nanoparticles was done using SEM (Quanta 250 FEG; FEI Company- Netherlands). Lyophilization was done by freeze dryer (CHRIST - Alpha 2-4 LSC basic- Germany) at −45 °C for 8 hours, the lyophilized sample was mounted onto aluminum specimen stub covered with a double-sided adhesive tape present on carbon disk.

##### 2.2.5.2. Differential Scanning Calorimetry (DSC)

The thermal properties of samples of CS, lecithin, TPP, GTE, physical mixture of all previously mentioned components, along with lyophilized unloaded and drug loaded CS-TPP nanoparticles were investigated using DSC (DSC- 60; Shimadzu- Japan). All samples were placed on an aluminum pan and were heated from a temperature of 25 to 400 °C and at a constant heating rate of 10 °C per min.

##### 2.2.5.3. Fourier transform infrared (FTIR) spectroscopy

Infrared spectra of each component as well as the final lyophilized formula was conducted to provide information about the functional groups present and interactions occurred in the elaboration of the optimized nanoparticles. Samples were prepared by grinding with potassium bromide individually then pressing them to form disks and FT-IR spectrophotometer (JASCO FTIR-6200, JASCO International Co., Ltd- Japan) was employed to record IR spectra (range 4000–400 cmˉ^1^).

##### 2.2.5.4. X-ray Diffraction (XRD) analysis

Determination of the crystalline or amorphous properties of CS, TPP, GTE and selected formula was analyzed using X ray diffractometer (D8 Bruker Co- Germany). The scanned diffraction angle 2θ ranged 2°- 50°, and the scan rate was 1° min − 1.

##### 2.2.5.5. *In vitro* release study

The release of GTE from the selected formula was tested using cellulose dialysis membrane method (Özcan et al., 2013). GTE loaded CS-TPP nanoparticles equivalent to 3 mg of GTE was placed in dialysis cellulose bag (12–14 kDa) previously soaked in phosphate buffer solution of pH 5.5. The release of equivalent amount of control samples (GTE solution (F*) and blank CS-TPP nanoparticles) was carried out simultaneously to exclude any factors that may affect the release from the nanoparticles. The dialysis bags were tied tightly from both ends and suspended in a beaker containing 50 mL of phosphate buffer solution at pH 5.5. The temperature was controlled at 37 ± 0.5 °C by incubating the beakers in thermostatistically controlled shaking water bath (Sci. FineTech. Model 220 V-50 Hz- Korea) at 100 rpm. An aliquot of 3 mL was drawn at definite time intervals (1, 2, 3, 4, 6, 8, 10, 12, 24 and 48 h) and an equal volume of dissolution medium was added to maintain constant volume and comply with sink condition. The concentration of the GTE in each withdrawn sample was quantified using UV spectrophotometer at 270 nm (Liu & Gao, 2009). Experiments were performed in triplicate and data were represented as mean ± standard deviation. The cumulative percentage released of GTE from nanoparticles was calculated.

##### 2.2.5.6. Release kinetics

*In vitro* release data was fitted to different kinetic models including zero order, first order and Higuchi, where the model exhibiting highest correlation coefficient (R^2^) was selected (Dash et al., 2010). The release data was also fitted into Korsmeyer-Peppas model to define the mechanism of drug release using the ‘N’ value estimated from the slope of the straight line (Yousry et al., 2017).

#### 2.2.6. *Ex vivo* study

##### 2.2.6.1. *Ex vivo* permeation

*Ex vivo* permeation study was conducted using female hairless Wistar albino rats (150 − 180 g). Hair present on the abdominal side was shaved and skin excised after sacrificing by cervical dislocation under light anesthesia, which was done after receiving the approval of the Research Ethics Committee, Faculty of Pharmacy, Cairo University (Cairo, Egypt) with protocol number PI (2158)) Skin collected was washed and kept hydrated in phosphate buffer solution pH 5.5 before conducting the experiment. A locally fabricated Franz-like diffusion cells composed of donor and receptor compartments with a diffusion surface area of 0.785 cm^2^ and receptor compartment capacity of 10 mL was employed in the experiment. Rats’ skin was placed between the two compartments with stratum corneum (SC) facing the donor compartment. A calculated amount of nanoparticles equivalent to 3 mg GTE were applied on the skin surface and sealed with a stopper to avoid its evaporation. Phosphate buffer solution, pH 5.5, was used in the receptor compartment along with magnetic stirrer bar to ensure constant agitation of the receptor media. The entire setup was placed in a water bath on a thermostatically controlled magnetic stirrer (Labnet – Accuplate PC 4200 – Mexico), and the temperature was maintained at 37 ± 0.5 °C. A volume of one milliliter sample was collected from the receptor compartment at specific time interval for a period of 48 hours; and was replenished with the same volume of fresh buffer to maintain sink condition throughout the experiment. The permeation study was also conducted for (GTE solution (F*) and blank CS-TPP nanoparticles) simultaneously to study the permeability enhancing activity of the selected formula. Samples withdrawn were analyzed using UPLC to identify the amount of caffeine, C, EC and EGCG permeated throughout the experiment. The experiment was performed in triplicate and results were expressed as mean ± SD (Puglia et al., 2016). Three permeation parameters were evaluated, namely, permeation flux, enhancement ration and lag time. The lag time was calculated as the intercept of the extrapolated linear part of the slope of the cumulative amount penetrated with the x-axis (Kilo et al., 2020).

The permeation flux was calculated using the following equation (Arafa et al., 2018):

(2) Jss=dQ/(dt*A)

The enhancement ratio was calculated using the following equation (Shakeel et al., 2009):

(3) ER=Jss of formulation/Jss of control

where:Jss = steady-state permeation flux (µg/cm^2^/h);A (cm^2^) = diffusional surface area; and(dQ/dt) = amount of drug permeating across skin per unit time at a steady state

##### 2.2.6.2. GTE deposition in skin

Penetration enhancing ability of the nanoparticles was determined at the end of the experiment through analyzing the amount of investigated components deposited in rats’ skin. Skin was removed, swabbed, and washed with a cotton dipped in phosphate buffer solution. The permeation area was excised, cut into small pieces, placed in 10 mL of the extracting solvent composed of 1:1 chloroform: acetonitrile and stirred at 500 rpm for 24 hours to maximize extraction of GTE components from skin (Hafner et al., 2011). Homogenization was carried out for 20 min, followed by centrifugation at 4 °C for 45 min at 10,000 rpm. The supernatant was filtered using filter syringe 0.22 μm pore size, evaporated to dryness using rotatory vacuum concentrator (Santoyo & Ygartua, 2000). Reconstitution of residue was done by addition of 1 mL distilled water and quantified for caffeine, C, EC, and EGCG using UPLC (Vyas et al., 2013; Hamishehkar et al., 2015).

#### 2.2.7. Statistical analysis

Each experiment was replicated three times and reported values were expressed as mean ± standard deviation. The statistical differences were performed in GraphPad Prism 6 software (GraphPad Software, La Jolla, CA) using one way analysis of variance (ANOVA) followed by Tukey’s test. Statistical significant difference was considered at *p*-value ≤ .05.

### 2.3. *In vivo* evaluation of the selected formula

*In vivo* study was carried out using 15 female albino Wistar rats weighing 180–200 g and were randomly distributed into three groups (*n* = 5) after the approval of the Research Ethics Committee at faculty of Pharmacy Cairo University: PI (2158)). Group (A) is the negative control group, group (B) receiving N8 equivalent to 50 mg GTE, and group (C) receiving equivalent amount of GTE suspension (DS). Each group was kept in an individual cage under well-defined and standardized condition where they were fed standard dry food and water ad libitum. They were kept for acclimatization for 1 week prior the start of the experiment. Group (B) and Group (C) received the aforementioned treatment twice daily topically, in the morning and evening, for 21 consecutive days.

#### 2.3.1. Histological examination

Histological evaluation of the subcutaneous tissue was carried out to assess and confirm the lipolytic effect of the optimized formula. After 21 days, skin samples of 1 cm^2^ area were collected and fixed in 10% neutral buffered formalin after scarification of rats by cervical dislocation under light anesthesia. The obtained tissue sections were collected on glass slides, deparaffinized and stained by hematoxylin & eosin stain (Bancroft & Gamble, 2008). The examination was done using Light microscope (Olympus BX43 light microscope fitted with DB27 camera-Japan). The dead bodies of the animals were frozen until being transported to be incinerated. Evaluation was done based on measuring three main parameters: (a) adipocyte perimeter, (b) adipocyte area and (c) fat layer thickness. Analysis of data was done using IBM SPSS statistics 20 software and applying one way ANOVA, where statistical difference was indicated when *p*-value < .05

#### 2.3.2. GTE deposition in skin following *in vivo* study

Amount of each investigated component was determined by collecting skin samples and extraction was done using chloroform: acetonitrile mixture. The organic solvent was evaporated using rotatory vacuum concentrator, then reconstituted by 200 μL deionized water, vortex for 2 min and injected in UPLC.

## 3. Results and discussion

### 3.1. GTE extraction procedure

First step of extraction preparation was drying and is considered a crucial step as it was important to reduce the moisture content to achieve the following: avoid microbial activity that might contaminate GTE, destroy the external waxy layer on the outer surface of the cells thus facilitate compounds to diffuse out during extraction, lastly and most importantly, to prevent oxidation of GTE components through inhibition of polyphenol oxidase enzymes. Twenty percent ethanol solution was found to be the most effective extraction solvent in extracting polyphenols (Rusak et al., 2008). Green tea extract powder obtained was dark brownish in color and crystalline appearance.

### 3.2. Phytochemical analysis

**Figure 1.**
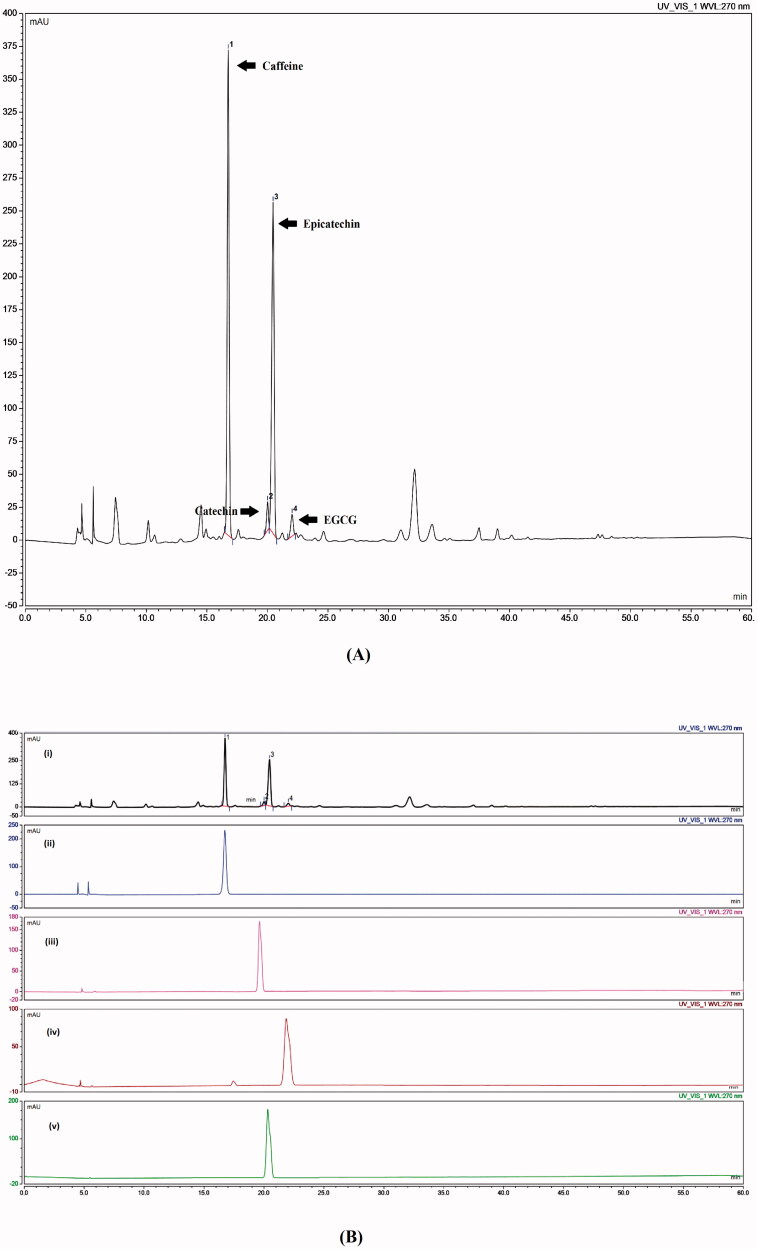
(A) GTE fingerprint, (B) GTE fingerprint and marker compounds (i) GTE, (ii) caffeine, (iii) catechin, (iv) epigallocatechingallate, and (v) epicatechin.

As a primary step for conducting the current study, chemical fingerprinting of GTE was conducted for the quality assurance of the used extract. That being said, fingerprinting is defined as a unique distinguishing pattern of multiple chemical markers inside a certain sample. Hence, chemical markers are ingredients present within a herbal product that participate in its medicinal activity and their presence act as an indicator for the quality of a medicinal plant (WHO, 2000). Consequently, in the present study, caffeine, C, EC and EGCG were adopted as the chemical markers of GTE. Figure 1(A) showed sharp and symmetric peaks with good baseline resolution. Moreover, each of the investigated component was identified on the chromatogram by correlating retention time of the authentic peaks against their corresponding peaks in GTE. The retention time (t_R_) of caffeine, C, EC, and EGCG present in GTE were 16.8, 19.72, 21.11 and 23.01 min respectively as demonstrated in Figure 1(B). The amount and the percentage of each of the desired component present in one milligram of GTE have been calculated using regression equation developed upon constructing calibration curves between the concentration and area under peak (AUP) of each of the investigated components. The amount (µg) present in one mg GTE was as follows: 58.9, 8.1, 3.2 and 35.7 µg/mg for caffeine, C, EC, and EGCG respectively.

### 3.3. Characteristics of the prepared GTE loaded CS-TPP nanoparticles

Two sets of groups have been prepared, first set N1-N3 had no additives while N4-N12 tested the effect of addition of lipid such as lecithin. Results shown in Table 2 revealed EE%, PS, PDI and ZP for each of the prepared formula.

**Table 2.** Mean EE%, PS, PDI and ZP of the prepared formulae.

Formulae	Mean EE (%) ± SD	Mean PS (nm) ± SD	Mean PDI ± SD	Mean ZP (mV) ± SD
N1	46 ± 1.79	180 ± 3.80	0.235 ± 0.012	21 ± 1.67
N2	26 ± 1.29	176 ± 2.16	0.249 ± 0.002	26 ± 1.11
N3	16 ± 1.53	159 ± 5.31	0.255 ± 0.006	33 ± 4.66
N4	49.22 ± 0.21	318.6 ± 7.64	0.416 ± 0.06	19.73 ± 2.02
N5	61.61 ± 0.12	412.9 ± 9.86	0.442 ± 0.048	14.33 ± 3.2
N6	71.94 ± 0.47	603.13 ± 17.79	0.765 ± 0.197	8.53 ± 3.61
N7	49 .18 ± 4.14	254.67 ± 8.50	0.230 ± 0.038	44.27 ± 1.78
N8	68.4 ± 1.88	292.6 ± 8.98	0.253 ± 0.002	41.03 ± 0.503
N9	74.11 ± 2.84	337.07 ± 7.64	0.416 ± 0.021	36.73 ± 1.514
N10	42.43 ± 5.46	194.00 ± 2.61	0.241 ± .016	30.60 ± 3.08
N11	46.81 ± 3.16	226.80 ± 6.16	0.170 ± 0.063	30.63 ± 0.86
N12	70.00 ± 5.94	319.60 ± 9.11	0.441 ± 0.02	28.30 ± 1.71

*Composition is given in Table 1.

#### 3.3.1. Absence of additives

Effect of CS: TPP mass ratio on GTE entrapment within nanoparticles was investigated, a significant decrease in the EE% was observed upon increasing mass ratio from 2:1 (N1) to 6:1 (N3) with *p*-value < .05. The decline in the entrapment efficiency at higher mass ratio was attributed to the insufficient amount of cross-linker in comparison to polymer available for entrapment. These results were comparable to Gan & Wang (2007), who suggested lower CS: TPP mass ratio favored a higher entrapment. PS and PDI have significantly decreased upon increasing mass ratio from 2:1 (N1) to 6:1 (N3) with *p*-value < .05. At lower mass ratio (N1), the availability of TPP relative to CS was high, thus forming more inter and intramolecular cross-linking resulting in large nanoparticles, while at higher ratios, 6:1 (N3), smaller particles were attained due to presence of insufficient amount of TPP incomparison to CS molecules available. These findings were consistent with Rampino et al and Konecsni et al (Konecsni et al., 2012; Rampino et al., 2013). It can be inferred that CS molecules at the mass ratio 2:1 were almost fully cross-linked and the excess TPP led to cross-linking between monodispersed particles into aggregates, resulting in larger particle size (Abosabaa et al., 2021). Increased PS could also be attributed to the accompanied increased in EE%. Regarding the ZP, a linear relation was wittnessed where increasing CS: TPP mass ratio resulted in a significant rise in the net positive charge. Lower ZP value present in (N1) was attributed to the excess negatively charged phosphate functional group of TPP, consequently decreased the presence of free unconjugated positive NH_3_^+^ group in CS-TPP complex, thus decreased the net positive surface charge (Pooja et al., 2014; Delan et al., 2020).

#### 3.3.2. Addition of lecithin

**Figure 2.**
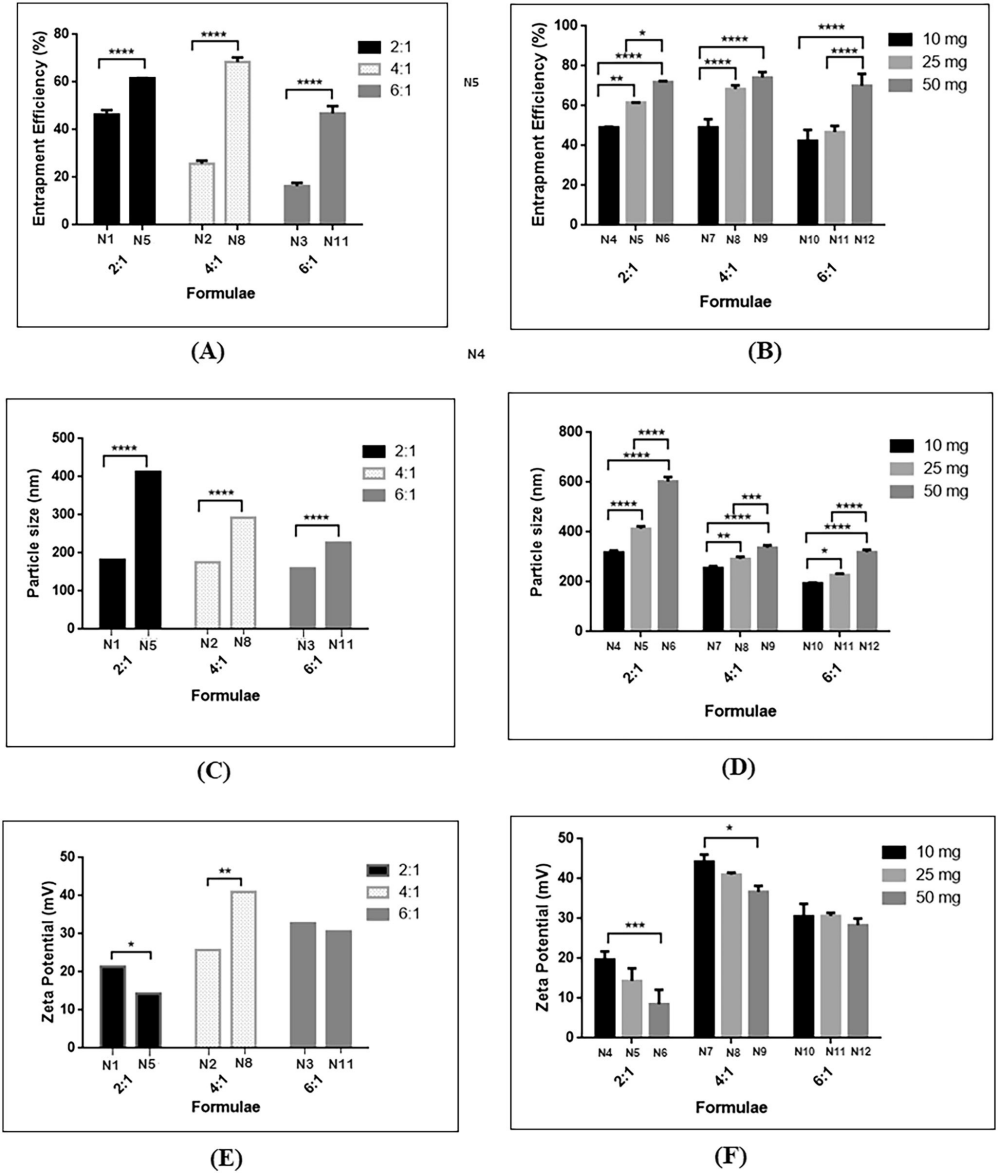
Effect of addition of lecithin at different CS: TPP mass ratios: (A) 25 mg lecithin on EE%, (B) 10, 25 and 50 mg lecithin on EE%, (C) 25 mg lecithin on PS, (D) 10, 25, 50 mg lecithin on PS, (E) 25 mg lecithin on ZP, and (F) 10, 25, 50 mg on ZP.

Hybrid lipidic nanoparticles were assembled via self-organizing interaction between CS and lecithin. Addition of lecithin during the formulation of GTE loaded CS-TPP nanoparticles have affected significantly increased the EE% with *p*-value < .05. This was demonstrated by addition of 25 mg lecithin in the three mass ratios 2:1 (N5), 4:1 (N8) and 6:1 (N11) as shown in Figure 2(A). Incorporation of lecithin led to escalation in EE% almost to the double in comparison to its absence owing to the presence of lipid in the core that provided structure integrity and prevented leakage of drug (Cheow & Hadinoto, 2011; Khan et al., 2019). Moreover, higher partitioning of drug to the lecithin core contributed in increasing EE% as reported previously (Bhatta et al., 2012). Effect of lower and higher amounts of lecithin, 10 mg and 50 mg respectively were also examined. As illustrated in Figure 2(B) increasing amount of lecithin led to a significant increase in the EE% in the three investigated mass ratios with *p*-value < .05. An apparent increase in the PS upon incorporation of 25 mg phospholipid in all three tested CS: TPP ratios was observed as shown in Figure 2(C). Similar results were observed upon the addition of different amounts of lecithin, namely 10 and 50 mg in each mass ratio as shown in Figure 2(D). This occurred due to presence of longer molecular chains of the phospholipid entangled together resulting in larger sized nanoparticles (Liu et al., 2016). It should be highlighted that regardless to the presence of lecithin, the mass ratio played an important role in PS as explained in section 3.3.1 where larger PS nanoparticles were attained at low mass ratios and smaller ones were obtained at higher mass ratio. Also, as reported by Perez-Ruiz et al. (2018), accompanied increase in GTE entrapment efficiency contributed in the increased PS. The polydispersity index of almost all formulae was below 0.5 which indicated good stability and homogeneity of nanoparticle dispersion (Manchanda & Sahoo, 2017). Regarding nanoparticles surface charge, it is worth mentioning that lecithin possesses an overall negative charge due to the presence of anionic phosphatidic acid. As shown in Figure 2(E), nanoparticles formed at mass ratio 2:1 and 6:1 underwent a significant decline of positive surface charge owing to the presence of lecithins’ anionic phosphatidic acid groups. Nanoparticles prepared at mass ratio 4:1 (N8) showed different behavior at which there was an increase in the ZP from 26 ± 1.11 mV to 41.03 ± 0.503 mV, which was in agreement with Liu et al (Liu et al., 2016), who noted an elevation in the positive value of ZP on increasing amount of lecithin. This phenomenon could be rationalized by the rearrangement of lipid into the core and presence of higher amounts of positively charged CS chains exposed on the surface. A pattern of decreasing ZP upon increasing amount of lecithin incorporated was noted in all mass ratios, as in Figure 2(F), this was in agreement with Khan et al. (2019) who observed shifting of ZP from a positive value to a negative value as the concentration of the lipids increased.

### 3.4. Selection and characterization of the selected optimal formula

Based on the aforementioned results, N8 was selected based on the EE%, PS and ZP reported in Table 2 for further investigation. The entrapment of each of caffeine, C, E and EGCG present in GTE in the selected optimum formula was quantified using UPLC which were 53.51 ± 0.767%, 52.48 ± 0.978%, 59.07 ± 0.078% and 96.47 ± 0.022% respectively.

#### 3.4.1. Microscopic evaluation

##### 3.4.1.1. Transmission electron microscope (TEM) analysis

**Figure 3.**
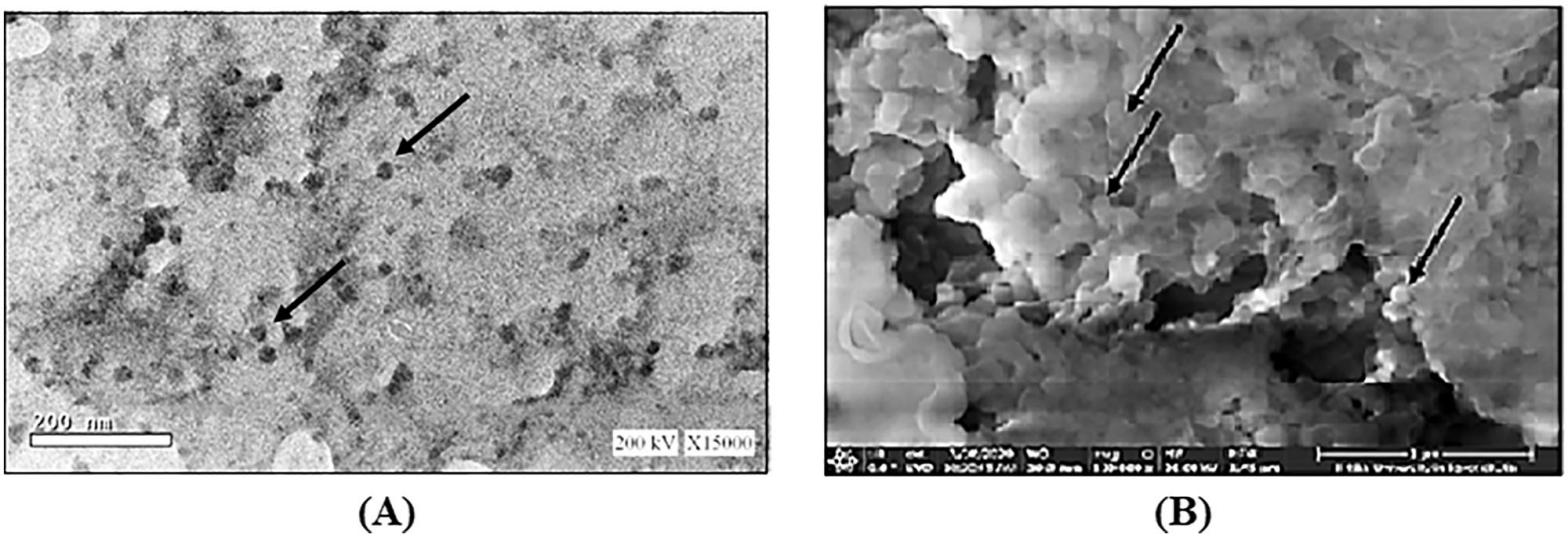
Microscopic examination: (A) TEM, and (B) SEM.

TEM analysis revealed homogenously spherical nanoparticles as shown in Figure 3(A). Moreover, aggregation was not observed possibly due to the high ZP which created steric hindrance (Sonvico et al., 2006; Chhonker et al., 2015). It is noteworthy that the size of the nanoparticles obtained by dynamic light scattering adopted by zetasizer were much larger in comparison to those detected by TEM. The latter is a multiangle measuring technique that measures the hydrodynamic radii of particles depending on the intensity of light scattering. In contrast, the TEM measures the actual size of the particles (Aktaş et al., 2005; Papadimitriou et al., 2008).

##### 3.4.1.2. Scanning electron micrsoscope (SEM) analysis

Morphological evaluation of the lyophilized sample using SEM showed appearance of uniform spherical structures of smaller PS ranging 250–300 nm with good homogeneity and aggregated due to freeze drying as presented in Figure 3(B) (Ilk et al., 2017).

#### 3.4.2. Differential scanning calorimetry (DSC) analysis

**Figure 4.**
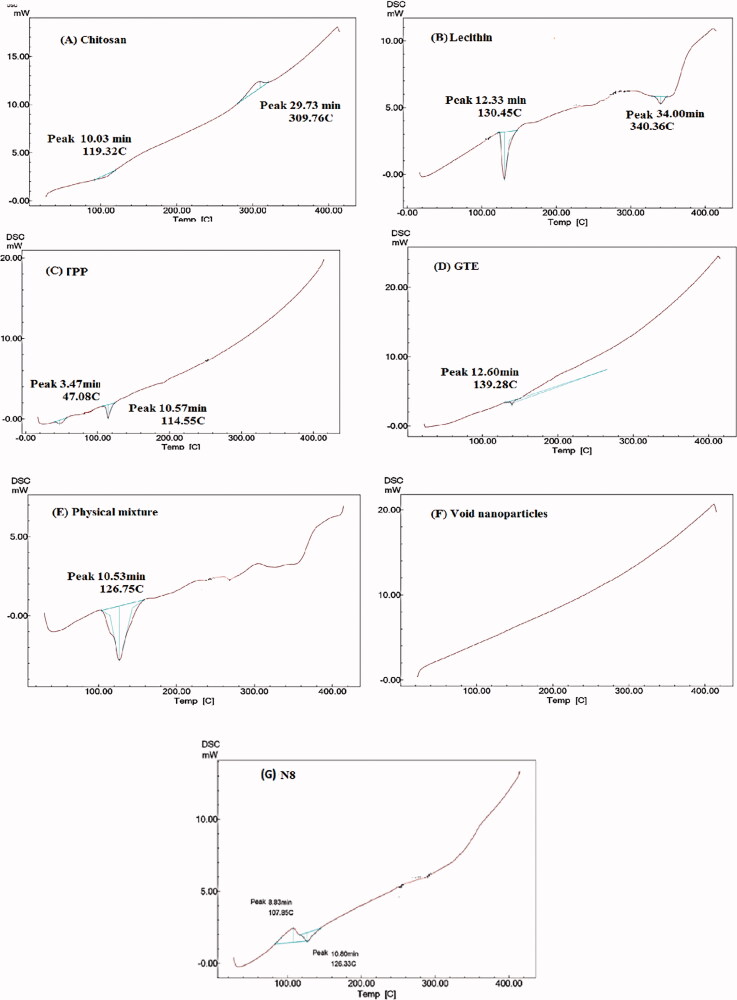
DSC thermogram of: (A) CS, (B) lecithin, (C) TPP, (D) GTE, (E) Physical mixture, (F) void nanoparticles and (G) GTE-loaded CS nanoparticles.

**Figure 5.**
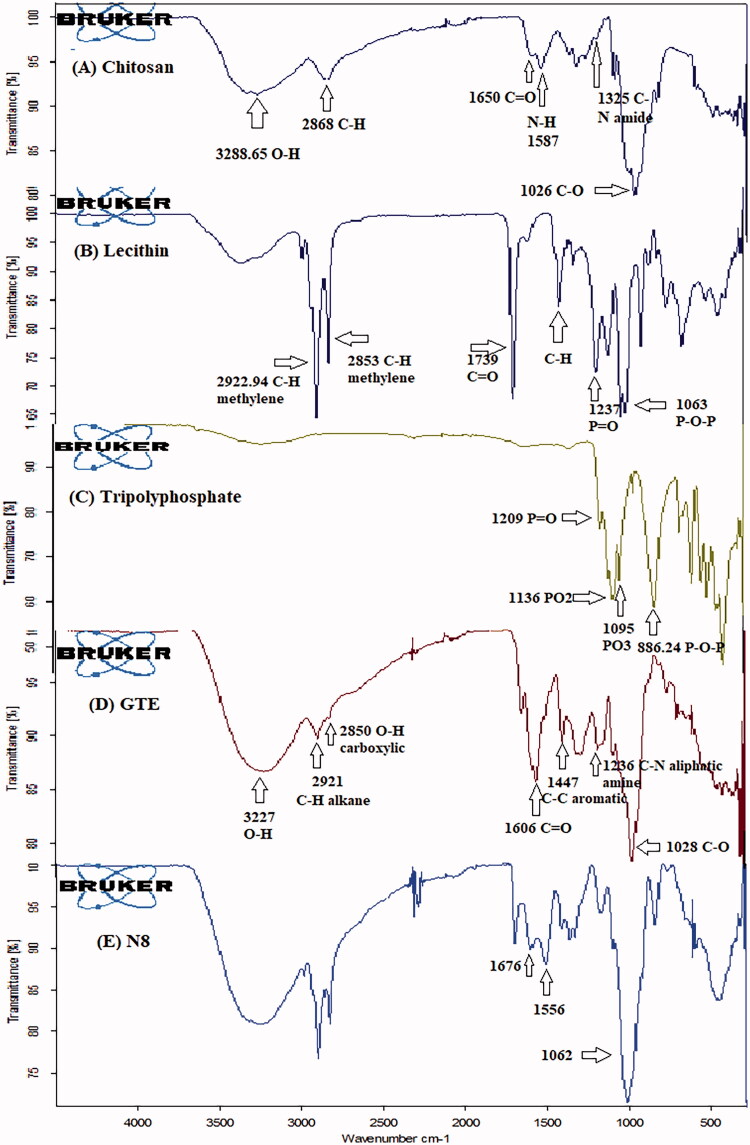
FTIR spectrum of: (A) CS, (B) lecithin, (C) TPP, (D) GTE, and (E) N8.

Thermal properties of optimized formula were examined using DSC, CS displayed an endothermic peak at 119.32 °C as shown in Figure 4(A), which was in harmony with Sabra et al. (2018), which was as a result of loss of moisture and melting of the CS (Khan et al., 2019). It was followed by well-defined exothermic peak at 302.76 °C indicating thermal decomposition which was in agreement with Arafa et al. (2020). Lecithin thermogram (Figure 4(B)) revealed two peaks, a sharp distinctive endothermic peak at 130.45 °C and a minor one at 340.36 °C respectively, which was consistent with Pathak et al. (2015). Two separate endothermic peaks were visible in TPP thermogram (Figure 4(C)), at 47.08 °C and 114.55 °C respectively in line with Manchanda & Sahoo (2017). Presence of two separate endothermic peaks suggested the presence of two different crystal structure. Thermogram of GTE shown in Figure 4(D) revealed a slight single endothermic peak at 139.28 °C indicating its crystallinity, this was close to the findings stated by Dzulhi et al. (2018). The physical mixture of the components exhibited a relatively broad peak around 126.75 °C (Figure 5(E)), indicating no interaction between components when physically mixed. On the contrary no peaks were detected in the void nanoparticles (Figure 4(F)) which suggest that there was a significant molecular interaction among the components. The endothermic peak of GTE in the loaded CS-TPP nanoparticles was not visible as shown in Figure 5(G), thus, confirming successful inclusion of GTE in N8 (Zhang et al., 2008).

#### 3.4.3. Fourier transform infrared (FTIR) spectroscopy analysis

FTIR spectrum of CS, lecithin, TPP, GTE and N8 is shown in Figure 5. Incomplete deacetylation of CS was confirmed by the existence of residual N-acetyl groups present at adsorption band 1650 cm^−1^ and 1325 cm^−1^ which corresponded to (C = O stretching in amide group, amide I vibration) and (C–N stretching of amide III), respectively as shown in Figure 5(A) (Ferreira Tomaz et al., 2018). A band corresponding to O–H and N–H stretching was observed around the region 3288.65 cm^−1^. A discrete absorption band was observed at 2868 cm^−1^ due to C-H stretching that are typically present in polysaccharide IR spectra. Primary amine functional group was detected due to presence of N-H bending at band 1587 cm^−1^. Regarding lecithin in Figure 5(B), C-H stretching vibration of methylene group was clearly spotted by the presence of intense absorption bands at 2922.94 cm^−1^ and 2853.27 cm^−1^. A strong signal also appeared at 1739 cm^−1^ corresponds to C = O stretching vibration, moreover, peaks at 1464 cm^−1^, 1237.27 cm^−1^, and 1063.87 cm^−1^ were due to C-H bending, P = O and P–O–C stretching vibration. These findings were also reported by Perez-Ruiz et al. (2018). Characteristic peak was visible in TPP spectra (Figure 5(C)) at 1209 cm^−1^ corresponding to P = O stretching. Additionally, intense absorption bands were evident at 1136 cm^−1^ and 1095 cm^−1^ owing to the presence of symmetric and asymmetric stretching vibration of PO_2_ and PO_3_ groups. Asymmetric stretching of P-O-P bridge was identified by the absorption band at 886.24 cm^−1^ (Ferreira Tomaz et al., 2018). Figure 5(D) show the IR spectrum of whole GTE extract, an immensely broad band was clearly apparent at 3227 cm^−1^ reflecting OH stretching vibrating ascribed to phenolic hydroxyl groups (Lisperguer et al., 2016). Absorption bands at 2921 and 2850 cm^−1^ were correlated to C-H vibrations of alkane and O-H of carboxylic acid respectively (Senthilkumar & Sivakumar, 2014). Flavonoid C = O functional groups and C-C of aromatic ring were recognized at 1606.3 cm^−1^ and 1447 cm^−1^ (Liang et al., 2011), while C-N of aliphatic amine and C-O stretching vibration appeared at 1236 cm^−1^ and 1028 cm^−1^ respectively (Senthilkumar et al., 2017). On investigating FTIR spectra of formulated nanoparticles – N8 (Figure 5(E)), successful interaction was elucidated due to disappearance of absorption band 1587 cm^−1^ corresponding to N-H primary amine of chitosan. Along this line, changes in the absorption bands of P = O group of lecithin and PO_2_ symmetric and asymmetric stretching of TPP present at 1237.27 cm^−1^ and 1136 cm^−1^ support aforementioned statement. Presence of two new peaks in 1676 and 1556 cm^−1^ was attributed to the phosphoric groups of TPP interacting with the ammonium groups of CS as described by Gokce et al. (2014).

#### 3.4.4. X Ray diffraction (XRD) analysis

**Figure 6.**
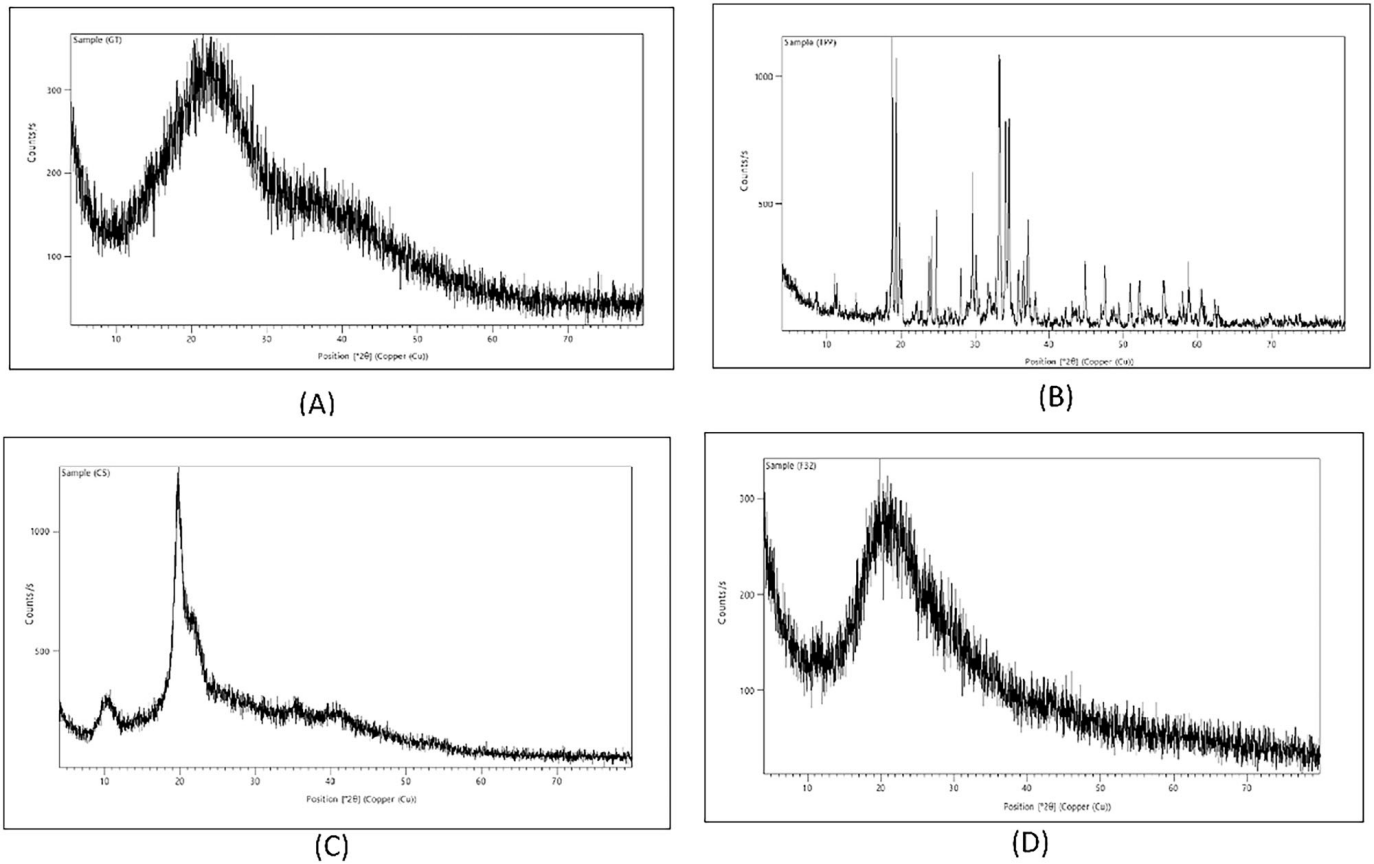
X-ray diffractogram of: (A) CS, (B) TPP, (C) GTE, and (D) GTE-loaded CS nanoparticles.

X-ray Diffraction was performed for CS, TPP, GTE and selected N8. CS diffractogram present in Figure 6(A) showed a wide peak at 2θ = 28.05°, while TPP in Figure 6(B), revealed characteristic peaks at diffraction angles of 2θ = 7°, 18.81°, 19.3788°, 24.1104°, 24.8053°, 29.6489°, 33.2765°, 34.1076°, 34.5673°, 37.1392°, 44.959° and 47.521°, showing dominant crystallinity. Diffractogram of GTE in Figure 6(C) showed two broad diffraction peaks at angles of 2θ = 10.208° and 19.77°. However, The XRD spectrum of N8 in Figure 6(D) exhibited non-crystalline behavior (amorphous nature), showing a very similar pattern to CS diffractogram, in which a broad peak was present at 2θ = 21.35°, no intense peaks of the GTE nor TPP were visible. Indicating that there has been a successful cross-linking between chitosan and TPP along with the dispersion of GTE within the nanoparticles (Arafa et al., 2020).

#### 3.4.5. *In vitro* release

**Figure 7.**
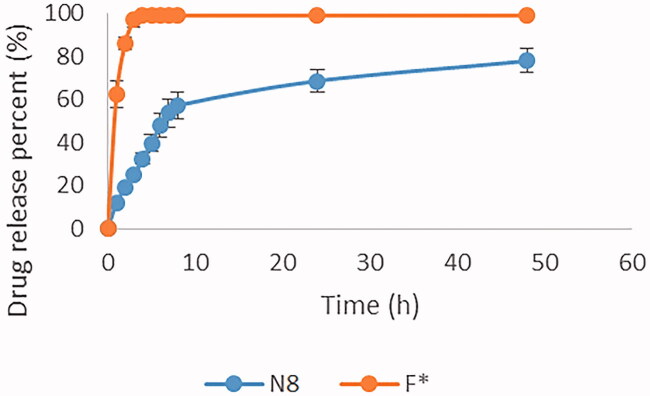
Percentage release of GTE from N8 and F* after 48 hours.

The cumulative percentage of drug release from N8 was compared to GTE solution (F*) throughout a period of 48 hour as shown in Figure 7. Hundred percent drug release from the GTE solution (F*) was achieved within 3 hours. In case of N8, a biphasic release profile followed by a sustained release pattern was observed reaching a 78% drug release by the end of the experiment. Initially, a rapid release of GTE was observed arising from the adsorbed GTE molecules present on nanoparticles surface as reported by Tran et al. (2015). This was followed by a sustained release pattern resulted from the presence of lecithin that may hinder the release of drug, as reported in previous study by Liu et al. (2016), who witnessed a decline in the percentage released from 50% to 20% upon doubling the amount of lecithin corresponding to CS, this was explained due to the increased amount of lecithin which resulted in higher electrostatic interaction with positively charged chitosan causing a slower release of the drug from the multilayered nanoparticles.

#### 3.4.6. Release kinetics

Drug release data where fitted into different kinetic models including zero order, first order and Higuchi model as shown in Table 3. Results revealed that optimum formula (N8) was best fitted in Higuchi diffusion model. These finding were in accordance Chadha et al. (2012). Mechanism of release was also investigated by applying Korsmeyer Peppas where the value of release component (N) of the selected formula was 0.5<*n* < 0.89 demonstrating non-Fickian diffusion. Such mechanism indicate that release was controlled by both erosion and diffusion mechanism. Primarily, polymer swell forming a gelatinous mass resulting in its relaxation (Arafa et al., 2020), followed by erosion. This was then followed by diffusion as the polymer became soluble and permeable (Motawi et al., 2017).

**Table 3.** Different release models and Korsemeyer Peppas model.

Formula	Model	**R** ²	Equation	Mechanism
N8	Zero order	0.8957	y = 0.0866x + 10.182	Higuchi diffusion (non-Fikian)
	First order	0.9287	y = 0.0006x + 1.9695	
	Higuchi	0.9629	y = 2.8842x − 10.373	
	Korsmeyer Peppas	0.9846	y = 0.7598x − 0.2999 **N = 0.7598**	

#### 3.4.7. Ex vivo study

##### 3.4.7.1. *Ex vivo* permeation

It is well known that the rate and extent of topical drug delivery considerably depend on the type and size of vehicles used. Furthermore, It has been reported that chitosan-lecithin nanoparticles enhance skin permeation (Fereig et al., 2020). For this reason, drug release properties of (N8) was achieved by examining two parameters, (a) rate of penetration of GTE through skin into the receptor compartment; (b) amount of GTE deposited in skin after completion of the experiment. In order to assess the supremacy of the selected formula as a carrier for topical delivery, the permeation and accumulation of drug across the skin from the nanoparticles were compared to GTE solution (F*). None of the investigated components were detected in the receptor compartment throughout the experiment in case of (F*). However, in case of (N8), only caffeine penetration appeared after 5 h with cumulative percentage of 12.4% in the receptor compartment. The percentage of permeated components of GTE depend on the molecular size and degree of hydrophobicity of each component. Caffeine has the smallest molecular weight, therefore can easily pass through skin into the donors compartment in comparison to larger molecular weight catechins. The permeation parameters tested included permeation flux (J, µg/cm^2^/h) was calculated as the slope obtained from the linear portion of the plot divided by the skin surface area (Adhikari et al., 2010), and enhancement ratio which is a ratio of the flux value of the chosen optimum formulation to the flux value of drug solution (Hussain et al., 2020; Altamimi et al., 2021). Formula (N8) showed a flux value of 0.1201 µg/cm^2^/h and an enhancement ratio of 0.12 which were relatively low along with sustained release profile with a lag time of 8.72 hr, which indicated that the selected formula was present at the site of action.

##### 3.4.7.2. GTE deposition in skin

The amount of each component in GTE loaded N8 retained in skin was quantified using UPLC in previously described method. Results shown in Table 4 revealed that retention of all components was achieved using CS-TPP nanoparticles with the addition of lecithin.

**Table 4.** Amount and percentage retention of each of the investigated components in skin after application of nanoparticles and GTE solution equivalent to 3 mg for 48 h.

Component	Amount of N8 applied on skin (µg)	Amount permeated to receptor compartment from N8 (µg)	Amount retained in skin from N8(µg)	Amount remained in N8 on skin surface (µg)	Percentage deposited in skin –N8 (%)	Percentage deposited in skin- GTE solution (%)
Caffeine	96	11.9	66.48	17.62	69.25 ± 3.63	0
C	12.81	0	9.26	3.55	72.28 ± 6.41	0
EC	5.58	0	5.244	0.336	93.98 ± 5.48	0
EGCG	103.2	0	29.11	74.09	28.21 ± 2.07	0

Epicatechin showed maximum skin retention while EGCG showed the minimum, this was attributed to the ocllusive properties nanoparticles which was enhanced as the PS was less than 400 nm as reported in the literature (Dzulhi et al., 2018). In that regard, the low flux values of caffeine along with the presence of the accumulated GTE components in skin following *ex vivo* permeation study, it can be stated that the prepared nanoparticles form a resevoir to prolong skin residence time and provide better treatment profile. High percentage skin deposition of caffeine, C and EC were attained due to their relatively lower molecular weight thus facilitating their skin penetration, while EGCG had the least deposition percentage due to presence of gallate group creating steric hindrence, which hindered skin penetration (Wisuitiprot et al., 2011). Moreoever, enzymatic hydrolysis of EGCG by esterase present in the SC as a potential degradation mechanism further clarify the low amount retained (Zillich et al., 2013).

#### 3.4.8. *In vivo* evaluation

##### 3.4.8.1. Histological characteristics

**Figure 8.**
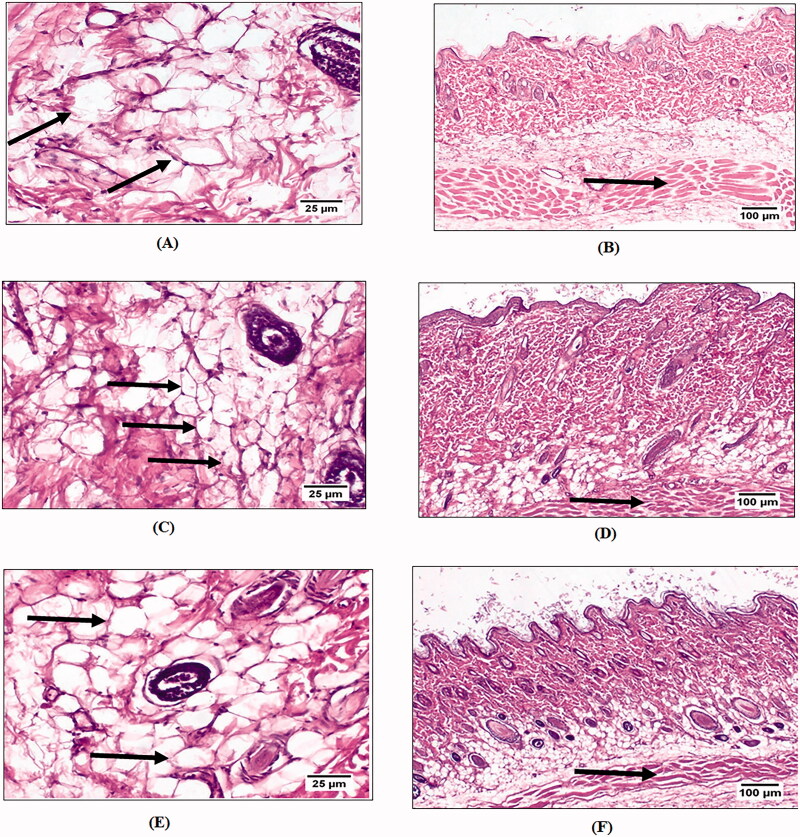
Control group showing: adipocytes (A), subcutaneous fat layer thickness (B), Groups B and C showing: significant reduction in area and size of adipocytes (C) and (E), a marked decrease in the subcutaneous fat layer thickness (D) and (F).

The potential anti-cellulite activity of pure GTE and N8 was evaluated by assessing adipocyte area, perimeter and subcutaneous fat layer thickness after application of GTE suspension and N8 for 21 days. Figure 8 shows the adipocytes and fat layer thickness stained with hematoxylin and eosin (H&E) for each group. The skin of all experimental groups exhibited normal histologic structure. The epidermal layer composed of stratified, squamous keratinized epithelium. The dermis consisted of dense and loose irregular connective tissue containing blood vessels, lymphatic vessels, and nerves. In addition, the skin appendages showed normal structure of hair follicles, sweat glands and sebaceous gland as shown in Figure 8(A). Safety of GTE and N8 as a topical delivery system was confirmed by the absence of major changes in the ultra-structure of skin morphology and epithelial cells. The dermis of negative control group showed abundant number of large diameter fat cells (Figure 8(A)) with thicker fat layer (Figure 8(B)) compared to other treated groups. After 21 days of treatment, group B receiving (N8) showed the lowest size of dermal adipocytes (Figure 8(C)) and fat layer thickness (Figure 8(D)) in comparison with other groups. Group C received GTE suspension (DS) showed a significant reduction in adipocyte area in Figure 8(E) and fat layer thickness in Figure 8(F) however it was not as effective as treatment using (N8). The effect of treatment using (N8) in comparison to DS on the adipocyte perimeter, area and fat layer thickness is illustrated in Table 5. Topical application of formula resulted in highest significant decrease in adipocytes area reaching up to a 46.96% reduction and 21.54% reduction in the fat layer thickness after three weeks with a *p*-value ≤ .05. Moreover, despite the lack of studies of using GTE solely as an anti-cellulite agent, these results proved its potentiality in ameliorating cellulite.

**Table 5:** Effect of topical treatment of group B and group C after 21 days on perimeter and area of the adipocytes as well as the thickness of fat layer compared to negative control group A.

Parameter	Group A	Group B	Group C
Adipocyte perimeter	73.11^d^ ±2.12	45.68^a^ ±1.22	54.14^b^ ± 1.62
Adipocyte area	338.68^c^ ± 17.1	179.61^a^ ±11.11	221.1^b^ ± 9.65
Fat layer thickness	199.4^c^ ± 5.24	156.43^a^± 3.98	182.43^b^± 5.86
% Decrease in adipocyte area	–	46.96^b^	34.71^a^
% Decrease in fat layer thickness	–	21.54^b^	8.51^a^

Data were expressed as mean ± SE. a, b, c and d within the same row indicate statistically significant difference at *P* ≤ 0.05. Group A is negative control, group B receiving N8, group C receiving GTE suspension (DS).

##### 3.4.8.2. GTE deposition in skin following *in vivo* study

Amount of GTE deposited in skin in group B and group C at the end of the study is shown in Table 6, expressed as mean ± SD. It is clear that there is a significant difference between the two groups showing significantly higher amount of caffeine and catechin components in group B retained in skin in comparison to group C, while EC and EGCG were present at relatively lower amounts. The results of the high deposition of caffeine and C were consistent with the findings of *ex vivo* studies conducted in section 3.4.7.2. These deposition values could be rationalized by their relatively smaller molecular weight which enhanced their permeation through the closely packed keratinocyte present in the stratum corneum (SC). The presence of higher amounts of EC and EGCG in skin after application of GTE suspension (DS) rather than nanoformulation could be explained by two theories; first, the phospholipid may have formed an extra lipid barrier on the skin surface; and second, high interaction may have existed between the lipids present in the formula with the components under question, which retarded their release from the nanocarrier leading to minimal skin deposition (Fang et al., 2005). At this juncture, it is worth clarifying that the amount of skin deposition of each component was zero after applying the GTE solution in the *ex vivo* skin permeation studies, this could be due their short time of application (48 hours). While, higher amounts of skin deposition of GTE components were observed after applying GTE suspension in the *in vivo* studies due to their application for a longer duration of 21 days.

**Table 6.** Amount (µg) of each component of GTE retained in skin after 21 days of topical application present in 0.785 cm*^2^* skin samples.

Component	Group B	Group C
Caffeine	36.52 ± 0.185	15.74 ± 0.24
C	33.21 ± 0.507	4.28 ± 0.04
EC	2.75 ± 0.35	4.04 ± 0.29
EGCG	0.62 ± 0.159	1.02 ± 0.23

Chitosan (CS) play a major role in increasing penetration of GTE into the deep layers of skin, the absorption promoting effect of CS improved adhesion between the formulation and the skin tissue. This adhesion was enhanced due to the attraction of oppositely charged CS with negatively charged skin surface, it loosened the intact accumulative structure of keratin in the SC through interactions with negatively charged SC cells and widened the tight junctions between epithelial cells in the skin (Lee et al., 2019; Enumo et al., 2020). Moreover, it also possesses transient effect on paracellular transport processes (Wang et al., 2008). Lecithin is another essential element in the nanoparticles. Lecithin was considered as penetration enhancers as this chemical showed a notable affinity to cellular membranes which led to an increased skin permeation with minimal adverse effects (Henmi et al., 1994), unlike other penetration enhancers such as organic solvents or fatty acids which usually generate skin irritation (Walters, 1989). This was consistent with our findings that group B received N8 showed normal histological structure as mentioned earlier. It enhanced drug penetration into skin by diffusing into the SC, disrupting its bilayer fluidity, loosening the lipid structure of the SC and providing impaired barrier function of the skin layers to the drug (Hamishehkar et al., 2013). Also, it has been reported that topical application of lecithin- containing formulation accelerate drug absorption by creating ‘lipid-enriched environment’ (Manconi et al., 2009; Fereig et al., 2021). Finally, GTE had the principle role in reducing adipocytes size and fat layer thickness. It acts through different pathways in reducing the subcutaneous fat tissues as mentioned previously. Moreover, it was indicated that GTE could transform white adipocytes to beige like adipocytes, which has higher lipid metabolism activity as it is rich with mitochondria (Sugiura et al., 2020).

## 4. Conclusion

Herbal extract, GTE incorporated in CS-TPP nanoparticles using lecithin was successfully prepared using ionic gelation technique. The optimal formulation selected was fully evaluated for its PS, PDI, ZP, EE%, microscopic analysis using TEM and SEM, thermal analysis using DSC, FTIR, XRD and *in vitro* release. *Ex vivo* permeation studies conducted showed high drug accumulation within skin with low flux values across skin proving successful permeability enhancing effect of the prepared formula. Further study using animal model were conducted and showed strong potential anti-cellulite activity of the optimal formula on subcutaneous adipocytes due to the dual effect of both CS and lecithin. Moreover, GTE had the principal role in reducing adipocytes size and fat layer thickness as it acts through different pathways in reducing the subcutaneous fat tissues. The satisfactory results obtained from the current study offer a new approach in the utilization of GTE loaded CS-TPP nanoparticles incorporated with lecithin for cellulite reduction.
